# Management status of patients with chronic kidney disease across medical specialties in Japan: a real-world data analysis

**DOI:** 10.1038/s41598-025-31735-2

**Published:** 2025-12-08

**Authors:** Shoichi Maruyama, Tetsuhiro Tanaka, Hirobumi Igawa, Mitsuru Hoshino, Shoichiro Inokuchi, Shuji Kaneko, Hirokazu Takaya, Asuka Ozaki

**Affiliations:** 1 Department of Nephrology, Nagoya University Graduate School of Medicine, Nagoya, Japan; 2 Department of Nephrology, Tohoku University Graduate School of Medicine, Sendai, Japan; 3 Cardiovascular, Renal and Metabolism, BioPharmaceuticals Medical, AstraZeneca, Osaka, Japan; 4 Evidence & Observational Research, Medical, AstraZeneca, Osaka, Japan; 5 Real World Evidence Division, Pharmaceutical Division, JMDC Inc., Tokyo, Japan

**Keywords:** Diseases, Health care, Medical research, Nephrology

## Abstract

Chronic kidney disease (CKD) contributes to poor renal and cardiovascular outcomes, necessitating early detection and intervention. The current study investigated the variability in CKD management practices across clinical specialties in Japan. Real-world electronic health records of 788,059 patients with suspected CKD between January 2004 and September 2021 were analyzed. Only 1.2% of patients received care in a nephrology department. However, nephrology involvement significantly increased the proportion of patients undergoing quantitative urine protein assessments and receiving a CKD diagnosis. This trend was evident in cardiology, urology, rheumatology, and endocrinology/metabolism departments, where nephrology involvement resulted in more frequent testing and earlier CKD diagnosis. Despite these benefits, the overall proportion of patients with nephrology involvement remains low, especially in the early stages of CKD. These findings highlight the considerable variability in CKD diagnosis and management approaches across clinical departments. Because quantitative urine protein assessment is critical for precise CKD diagnosis and patient care, strategies such as promoting interdepartmental collaboration with nephrology departments and establishing standardized diagnostic protocols tailored to individual specialties and facilities should be prioritized. Implementing such facility- and specialty-specific measures is crucial for enhancing early CKD detection and intervention, ultimately improving clinical outcomes in patients at risk of CKD progression.

**Supplementary Information:**

The online version contains supplementary material available at 10.1038/s41598-025-31735-2.

## Introduction

Chronic kidney disease (CKD) affects life expectancy and quality of life substantially. Even in its early stages CKD is associated with increased risks of renal events, cardiovascular complications, progression to end-stage renal disease (ESRD), and premature mortality. Early clinical intervention significantly improves patient outcomes^1,2^.

In our previous paper^3^, real-world data from electronic health records was used to evaluate the prevalence and characteristics of individuals with suspected CKD in Japan. We found that 57.5% of patients had undergone semi-quantitative urine protein testing, whereas only 6.9% were diagnosed with CKD. In a subsequent investigation^4^, we assessed the prognosis of patients with CKD stratified in accordance with the Kidney Disease: Improving Global Outcomes (KDIGO) risk classification^2^. In this analysis, the risk of major adverse cardiovascular events increased independently with both declining estimated glomerular filtration rate (eGFR) and increasing urine protein, even in early KDIGO risk classification stages.

These findings underscore the importance of promoting urine protein measurement to facilitate timely and accurate CKD diagnosis. Given the potential variability in CKD management across clinical specialties, a detailed investigation of practices within individual clinical departments is warranted. The current study investigated proportions of urine protein measurements and CKD diagnoses in clinical departments in Japan.

## Methods

The study design, database source, categorization via KDIGO risk classification, and methodology used for diagnostic detection in the present study have been previously described^3^. A detailed flow diagram summarizing patient selection and exclusion is provided in the prior publication^3^. The current study is an ad-hoc analysis based on an observational study using an identical population and dataset^3^. Briefly, the RWD database maintained by the Health, Clinic, and Education Information Evaluation Institute (Kyoto, Japan) and JMDC Inc. (Tokyo, Japan) was used. This database incorporates inpatient and outpatient electronic medical records from approximately 200 medical institutions in Japan. Notably, most of the data originated from hospitals, with only 10 clinics included in the participating institutions. Patients with two or more eGFR measurements below 90 mL/min/1.73 m², spaced more than 90 days apart but less than 360 days apart, were identified, consistent with the CKD definition requiring persistent eGFR abnormalities for at least 3 months. The identified population is considered a suspected CKD population because individuals with 60 ≤ eGFR < 90 alone do not meet the formal criteria for CKD but may be at risk or represent early-stage CKD. eGFR values were calculated using a formula developed for the Japanese population, based on serum creatinine, age, and sex^5^. The date of the first measurement was defined as the index date. The index dates were between January 1, 2004, and September 30, 2021. The following patients were excluded: (i) aged < 18 years; (ii) those without a 360-day look-back period; (iii) with erroneous death records prior to the index date; and (iv) index date not between January 1, 2004, and September 30, 2021^3^. Urine protein measurement was identified for each patient within the look-back period (Supplementary Figure S1). The study population was divided into five eGFR stages based on the eGFR levels in mL/min/1.73 m² during the look-back period; G2 (≥ 60), G3a (< 60), G3b (< 45), G4 (< 30), and G5 (< 15). Patients with semi-quantitative (i.e., dipstick) or quantitative urine albumin/protein results on the same date as the eGFR measurement were further categorized into the albuminuria stages: A1, A2 and A3, and other patients were categorized as having ‘unknown’ urine protein (Supplementary Table S1). If more than one value was obtained during the observation period, the earliest value was used.

No imputation was performed for clinical department or laboratory findings in this study. CKD diagnosis was defined as the presence of at least one CKD-related ICD-10 code within a period extending from 360 days before to 90 days after the index date (Supplementary Table S2). Comorbidities were defined by the presence of ICD-10 codes at or before the index date.

Clinical department information recorded in the database was used to classify medical specialties (Supplementary Table S3). Departmental attributes were retrieved from the DPC-FF1 data during the look-back period. Patients with nephrology involvement were counted separately within each non-nephrology department as co-managed cases. Sensitivity analyses were performed when assessing nephrology involvement in urine protein testing and CKD diagnosis, using data limited to the facilities with nephrology departments.

The study was approved by the Ethics Committee of the Research Institute of Healthcare Data Science (approval number RI2021018), and because only de-identified data were used, the requirement for informed consent was waived. All methods were performed in accordance with the relevant guidelines and regulations.

## Results

### Demographic distribution of patients with suspected CKD

**Fig. 1:**
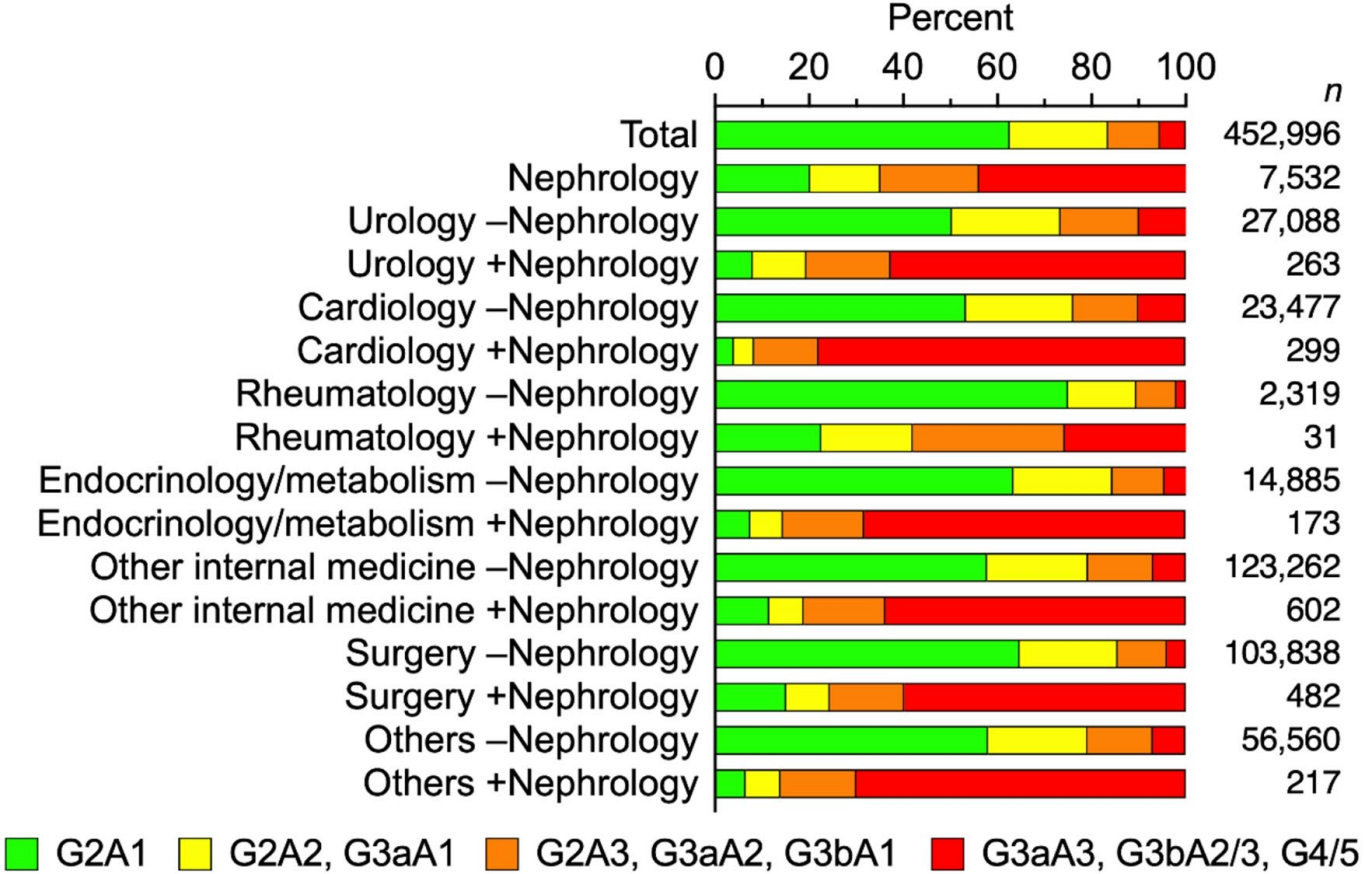
KDIGO risk classification of patients with suspected CKD in the clinical departments. ‘–Nephrology’ indicates cases without co-management of the nephrology department. ‘+Nephrology’ indicates cases co-managed with the nephrology department. Detailed data are shown in Supplementary Table S4.

A total of 788,059 patients were included in the study (Table 1), of which a nephrology department was involved in the care of 9,104 (1.2% of the total population), either as the primary provider or with the involvement of other clinical departments. The respective numbers of patients who visited urology, cardiology, rheumatology, and endocrinology/metabolism departments were 36,479, 59,499, 3,092 and 18,653, respectively. The proportions of patients that received nephrology across these departments ranged from 0.7% to 1.0%, with no marked differences between specialties. In every department, the proportion of patients receiving care involving nephrology increased with eGFR stage and KDIGO risk classification (Fig. 1, Supplementary Table S4).

**Table 1:** Characteristics of patients with suspected CKD in the clinical departments.

Department		Population <% of total> [% within ± Nephrology]	*n* of patients (% in the population)				
			Male	Age (mean ± SD)	Comorbidity of heart failure	Comorbidity of hypertension	Comorbidity of diabetes mellitus
Total		788,059	402,720 (51.1%)	63.9 ± 15.5	121,635 (15.4%)	270,304 (34.3%)	216,728 (27.5%)
Nephrology		9,104 < 1.2%>	5,306 (58.3%)	63.8 ± 16.8	3,428 (37.7%)	5,936 (65.2%)	3,982 (43.7%)
Urology	–Nephrology	36,137[99.1%]	27,123 (75.1%)	69.3 ± 12.9	4,161 (11.5%)	11,113 (30.8%)	9,279 (25.7%)
	+Nephrology	342[0.9%]	236 (69.0%)	67.4 ± 14.7	135 (39.5%)	219 (64.0%)	153 (44.7%)
Cardiology	–Nephrology	59,061[99.3%]	34,109 (57.8%)	69.4 ± 13.1	30,737 (52.0%)	39,828 (67.4%)	24,825 (42.0%)
	+Nephrology	438[0.7%]	295 (67.4%)	70.8 ± 13.3	334 (76.3%)	370 (84.5%)	280 (63.9%)
Rheumatology	–Nephrology	3,060[99.0%]	832 (27.2%)	60.3 ± 16.0	660 (21.6%)	692 (22.6%)	1,119 (36.6%)
	+Nephrology	32[1.0%]	12 (37.5%)	56.9 ± 19.0	17 (53.1%)	24 (75.0%)	22 (68.8%)
Endocrinology /metabolism	–Nephrology	18,465[99.0%]	9,510 (51.5%)	59.6 ± 14.1	2,524 (13.7%)	8,232 (44.6%)	13,410 (72.6%)
	+Nephrology	188[1.0%]	119 (63.3%)	64.1 ± 14.2	91 (48.4%)	164 (87.2%)	167 (88.8%)
Other internal medicine	–Nephrology	236,630[99.7%]	119,977 (50.7%)	65.8 ± 15.4	41,507 (17.5%)	93,664 (39.6%)	79,991 (33.8%)
	+Nephrology	778[0.3%]	487 (62.6%)	66.1 ± 15.9	367 (47.2%)	580 (74.6%)	421 (54.1%)
Surgery	–Nephrology	201,336[99.7%]	86,475 (43.0%)	65.3 ± 15.0	26,266 (13.0%)	69,703 (34.6%)	54,738 (27.2%)
	+Nephrology	668[0.3%]	389 (58.2%)	63.0 ± 17.3	345 (51.6%)	476 (71.3%)	350 (52.4%)
Others	–Nephrology	107,653[99.7%]	52,530 (48.8%)	66.9 ± 15.8	16,642 (15.5%)	45,597 (42.4%)	32,097 (29.8%)
	+Nephrology	318[0.3%]	194 (61.0%)	69.9 ± 16.4	187 (58.8%)	259 (81.4%)	185 (58.2%)

‘–Nephrology’ indicates cases without co-management of the nephrology department. ‘+Nephrology’ indicates cases co-managed with the nephrology department.

The proportions of male patients were highest in urology (75.0%) and lowest in rheumatology departments (27.3%). Mean patient age did not differ significantly across the departments, irrespective of nephrology involvement. Heart failure prevalence was high in cardiology (52.2%) and nephrology departments (37.7%), and in patients co-managed by both cardiology and nephrology departments, the prevalence was 76.3%. Hypertension was present in ≥ 60% of patients in both nephrology and cardiology, whereas the overall prevalence of hypertension in all patients was 34.3%. The prevalence of diabetes mellitus was 27.5%, second only to hypertension. In department-level analyses, the prevalence was higher with nephrology co-management than without (urology 25.7% vs. 44.7%; cardiology 42.0% vs. 63.9%; rheumatology 36.6% vs. 68.8%; endocrinology/metabolism 72.6% vs. 88.8%; Table 1).

The median HbA1c was 5.7% overall. Across departments, HbA1c was highest in endocrinology/metabolism and was similar regardless of nephrology involvement (urology 5.8% vs. 5.7%; cardiology 5.8% vs. 5.7%; rheumatology 5.7% vs. 5.6%; endocrinology/metabolism 6.8% vs. 6.7%). The median LDL-C was 114.0 mg/dL overall. Across departments, LDL-C values were similar and were lower when nephrology was involved (urology 112.0 vs. 98.0 mg/dL; cardiology 104.0 vs. 93.0 mg/dL; rheumatology 113.0 vs. 106.5 mg/dL; endocrinology/metabolism 110.5 vs. 99.5 mg/dL) (Supplementary Table S5).

Overall, angiotensin-converting enzyme inhibitors (ACEi), angiotensin receptor blockers (ARB), mineralocorticoid receptor antagonists (MRA), sodium–glucose cotransporter-2 inhibitors (SGLT2i), and statins were prescribed in 3.1%, 12.2%, 2.5%, 0.5%, and 10.6% of patients, respectively. Use of of ACEi/ARB, MRA, and statins was generally higher with nephrology involvement, whereas SGLT2i use was uncommon and largely limited to endocrinology/metabolism (Supplementary Table S5).

### Urine protein measurement

A semi-quantitative urine test using a dipstick was performed in 63.2% of the total population, resulting in 70.9% negative (–) and 29.1% positive (± to 4+) results (Supplementary Table S6). In nephrology departments, 85.2% of patients underwent a semi-quantitative test, with 33.5% testing negative and 66.5% testing positive. Respective testing proportions in cardiology, rheumatology and endocrinology/metabolism departments without nephrology involvement were 49.1%, 78.2%, and 82.7%. In contrast, with nephrology involvement the respective testing proportions were 76.3%, 96.9%, and 95.2%.

**Fig. 2:**
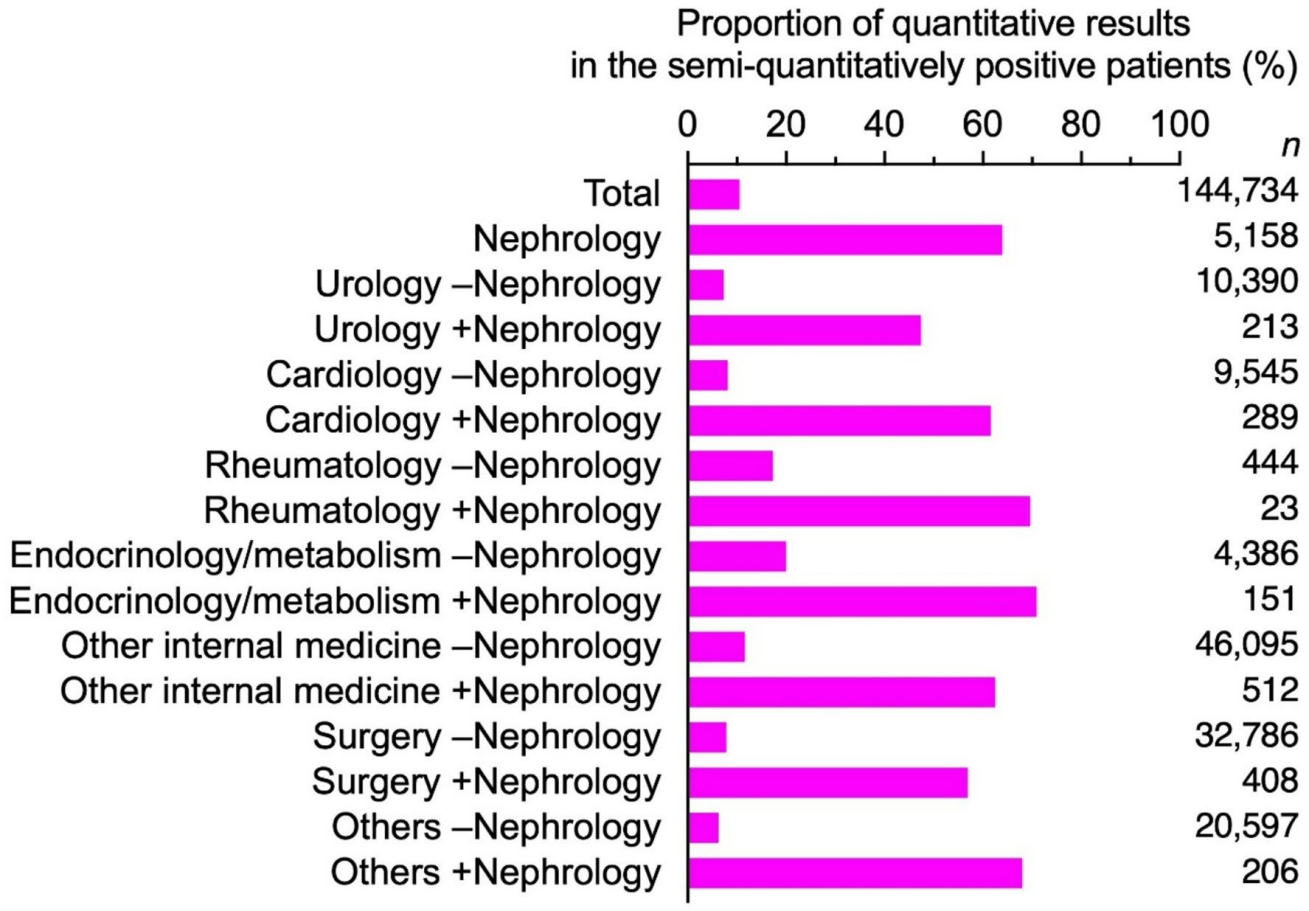
Proportion of quantitative results in the semi-quantitatively positive patients. ‘–Nephrology’ indicates cases without co-management of the nephrology department.‘+Nephrology’ indicates cases co-managed with the nephrology department. Detailed data are shown in Supplementary Table S6.

A similar trend was also observed with respect to quantitative urine testing. Of patients with positive semi-quantitative results, 63.9% underwent quantitative urine testing in a nephrology department. In urology, cardiology, rheumatology, and endocrinology/metabolism departments without nephrology involvement, the respective proportions were 7.3%, 8.1%, 17.3%, and 20.0%. With nephrology involvement, the respective proportions were 47.4%, 61.6%, 69.6%, and 70.9%, respectively (Fig. 2, Supplementary Table S6). In sensitivity analysis restricted to facilities with an in-house nephrology department (Supplementary Table S7), the direction and magnitude of associations were consistent with the main analysis.

### CKD diagnosis

**Fig. 3:**
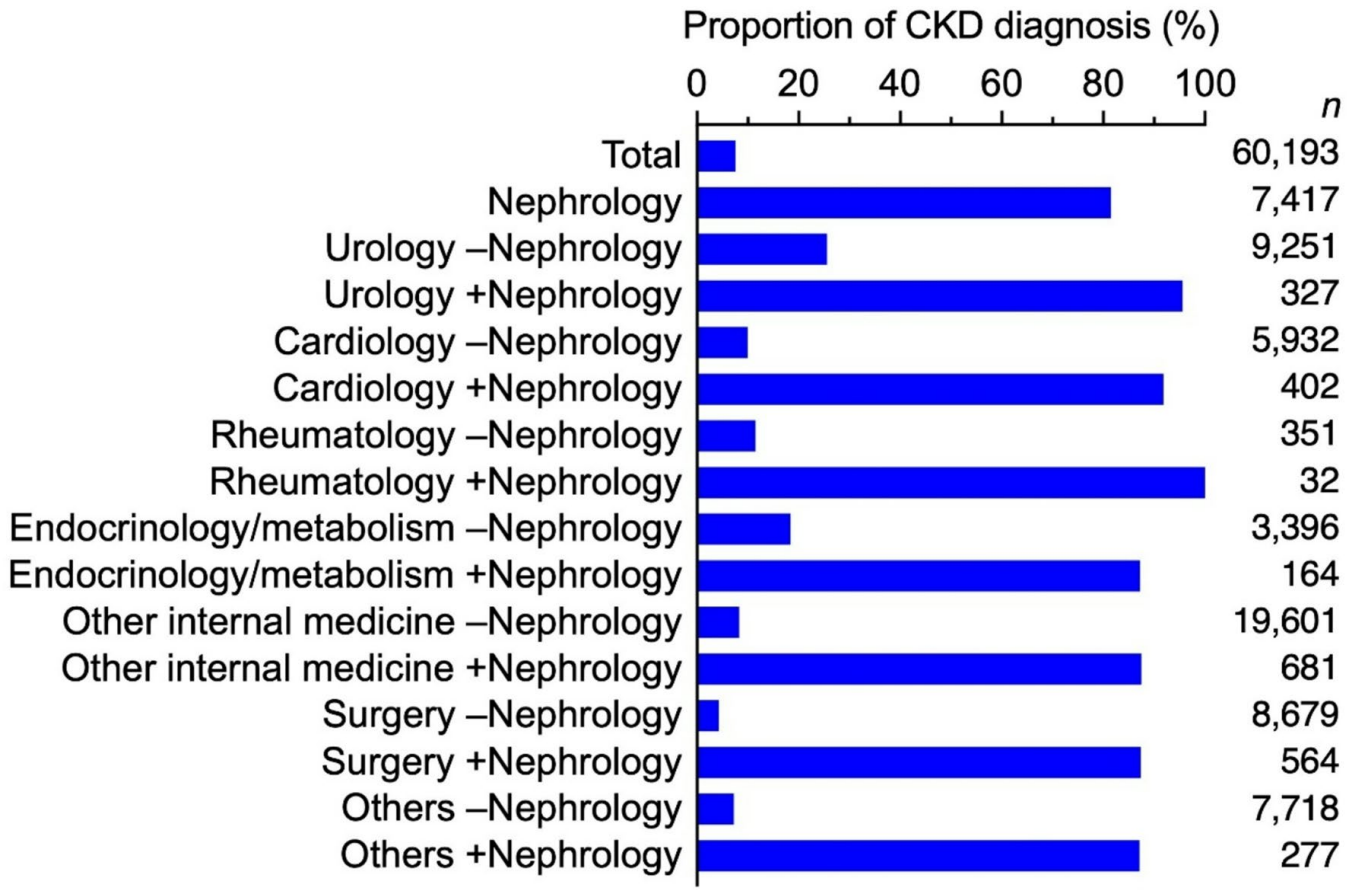
Proportion of CKD diagnosis in the clinical departments. ‘–Nephrology’ indicates cases without co-management of the nephrology department. ‘+Nephrol’ indicates cases co-managed with the nephrology department. Detailed data are shown in Supplementary Table S8.

Proportions of patients diagnosed with CKD were evaluated within each KDIGO risk classification across all clinical departments (Fig. 3, Supplementary Table S8). In nephrology departments, the overall proportion of CKD diagnoses was 81.5%, but it exceeded 90% in patients with advanced eGFR stages (G3b and above). The respective proportions of CKD diagnoses without nephrology involvement in urology, cardiology, rheumatology, and endocrinology/metabolism departments were 25.6%, 10.0%, 11.5%, and 18.4%. The respective proportions with nephrology involvement were 95.6%, 91.8%, 100%, and 87.2%. Across all KDIGO risk classifications and eGFR stages, the diagnostic rate was consistently higher when nephrology was involved. In sensitivity analysis restricted to facilities with an in-house nephrology department (Supplementary Table S9), the trends were consistent with the main analysis.

## Discussion

This study is the first to investigate the management status of patients with CKD across different clinical departments in Japan by analyzing the proportions of patients who underwent urine protein testing and were diagnosed with CKD in each type of department. Overall, the number of patients who visited a nephrology department was small, regardless of their involvement with other departments. However, when nephrology was involved, the proportion of patients with quantitative urine protein testing and a CKD diagnosis was higher. Across departments, semi-quantitative testing was commonly performed even without nephrology involvement (Supplementary Table S6). Yet, after a positive semi-quantitative result (± to 4+), quantitative confirmation was seldom obtained in non-nephrology settings but markedly more frequent with nephrology involvement (Fig. 2, Supplementary Table S6). This implementation gap likely leads to missed opportunities for accurate KDIGO staging and timely intervention. To ensure appropriate management of CKD, it is necessary to increase the implementation of quantitative urine protein testing and the diagnosis. To achieve this, facility-specific measures such as promoting nephrology involvement or encouraging testing and diagnosis within individual departments should be considered.

In this study, only 1.2% of patients received care from a nephrology department. The database used in this analysis only captured involvement within the same institution, and not across multiple facilities, potentially leading to underestimation. Another possible reason for the low nephrology involvement is that a large majority of the study population (82.6%) was classified as eGFR stage G2 (Supplementary Table S4). When focusing on more advanced CKD, nephrology involvement increased substantially; for example, among patients classified as G4, the proportion of co-management with nephrology departments rose to 15.6%. This proportion is broadly consistent with previous reports from the United States and Europe^6–8^. Nevertheless, the analysis suggests that many patients with CKD may not be adequately followed up despite their clinical conditions.

Our previous study^3^ indicated that quantitative evaluation of urine protein is essential for accurate CKD diagnosis, and the current analysis supports this by stratifying results across clinical departments. In nephrology departments, the proportions of both semi-quantitative and quantitative urine protein testing were high, and CKD diagnoses were frequent, even in early stages. Semi-quantitative urine protein tests were performed in urology, rheumatology, and endocrinology/metabolism departments without nephrology involvement, but the proportions of quantitative testing and CKD diagnoses remained low. When the nephrology department was involved, both proportions were higher. Similarly, in cardiology departments the proportion of semi-quantitative testing was low without nephrology involvement, but both quantitative testing and CKD diagnoses increased markedly when a nephrology department was involved. This trend of increased quantitative urine protein testing and CKD diagnoses with nephrology involvement was evident in the early stages of CKD. The Clinical Practice Guidebook for the Diagnosis and Treatment of CKD 2024 (Japanese), published by the Japan Society of Nephrology, emphasizes that even mild urine protein—such as a dipstick result of (±)—is an indication of CKD. When urine protein is detected using semi-quantitative tests, quantitative assessment of urine protein and albuminuria is recommended. Appropriate testing for the presence of mild urinary abnormalities is essential for the diagnosis and management of CKD. Therefore, facility-specific measures are necessary when mild urine protein is detected, to facilitate early CKD diagnosis and intervention.

The overall proportion of semi-quantitative urine protein tests was 63.2%, which was higher than previously reported in Japan^9^, and notably the proportions were 85.2% in the nephrology department and approximately 80% in urology, rheumatology, and endocrinology/metabolism departments. These higher proportions may be attributed to the characteristics of the patients analyzed and the databases used, as the analysis specifically focused on patients whose eGFR values were measured more than twice within a relatively short period. Additionally, more than 90% of the patients included received medical care at large hospitals with over 100 beds (Supplementary Table S10), facilities where semi-quantitative urine protein testing using dipsticks could be routinely performed compared to smaller facilities such as clinics.

The prevalence of heart failure was 52.2% in patients who visited cardiology departments, and increased to 76.3% in those with concurrent nephrology involvement. In patients who visited a nephrology department, the prevalence of heart failure reached 37.7%. The prevalence of hypertension, a common risk factor for both heart failure and CKD, exceeded 60% in both nephrology and cardiology departments. These data implicate cardio-renal syndrome^10^. Accordingly, routine renal function testing and urine protein assessment within heart failure care pathways may be warranted, and closer collaboration between cardiology and nephrology could be beneficial. Diabetes mellitus was the second most prevalent comorbidity after hypertension and was more common in groups with nephrology involvement (Table 1), likely reflecting referral/severity bias and a higher comorbidity burden. Given the close link between diabetes, CKD progression, and albuminuria, ensuring a reliable transition to quantitative assessment after a positive result on semi-quantitative urine protein tests is important across departments, irrespective of nephrology involvement. Glycemic and lipid parameters were broadly similar across departments, and medication patterns suggested more intensive risk-factor management where nephrology was involved, although outcome effects were not assessed. Notably however, 438 patients (0.7%) had both cardiology and nephrology involvement, and this proportion is low considering the high prevalence of CKD in patients with heart failure. This may be attributed to the limitations of the dataset, which captured nephrology department involvement only within individual facilities, and not across multiple institutions. Moreover, the low proportion of quantitative urine protein testing in cardiology departments could be explained by nephrology department at external facilities that manage kidney function. Additionally, low nephrology department involvement, even at advanced eGFR stages (G4 and G5), may also have resulted from this dataset limitation. With respect to nephrology department involvement, the proportions of urine protein testing and CKD diagnoses exhibited similar trends in a sensitivity analysis that focused on facilities with nephrology departments (Supplementary Tables S7 and S9), indicating that the observed differences are robust and not solely attributable to facility composition. Consequently, the extent of quantitative testing in non-nephrology departments, and the level of nephrology involvement at advanced eGFR stages may be biased downward. Accordingly, statements about low overall nephrology involvement should be interpreted as minimum estimates within single institutions, rather than definitive figures for the broader healthcare system.

The present study had some limitations. The real-world database used only included data collected from contracted medical institutions, it was not compiled via random sampling. Therefore, the data used in this study do not fully represent clinical practice across Japan. In particular, only 10 clinics (with fewer than 20 beds) were included in the database, despite the existence of approximately 100,000 clinics nationwide. Because primary-care clinics are underrepresented, our findings mainly reflect hospital-based practice and may not generalize to outpatient settings. The overall proportion of semi-quantitative urine protein tests was higher than that previously reported in Japan^9^, likely reflecting biased patient sampling from larger hospitals, and inclusion criteria requiring at least two eGFR measurements within a limited timeframe. Furthermore, the database relied on DPC claims and electronic medical records, the reliability of which depends on the accuracy of the original corresponding records. Because the database does not capture differences in measurement methods, laboratory results were not standardized and may have measurement bias. Patients could not be followed up after transfer to other healthcare facilities, and there is a possibility that patients re-entered the database after initially dropping out. Additionally, we could not exclude transient (non-pathological) elevations in urine protein, and semi-quantitative tests can be affected by urine concentration and pH and has limited sensitivity for low-grade albuminuria. These may have led to misclassification, especially in early CKD. As this is a retrospective observational study, the associations reported are not causal. They may be influenced by referral/selection bias and unmeasured confounders. Finaly, it should be noted that CKD was identified using ICD-10 codes and may not always align with KDIGO biochemical criteria registered as a disease name mainly for insurance purposes, which may not directly reflect the level of awareness among healthcare professionals regarding CKD management, and may introduce coding-related misclassification.

Future work should link these results to clinical outcomes such as CKD progression, cardiovascular events, and mortality.

## Conclusion

This study identified significant variability in CKD management practices across different clinical specialties in Japan. Although nephrology department involvement markedly improves the frequency of quantitative urine protein assessments and CKD diagnosis, such involvement remains limited, particularly in the early stages of CKD. Given that quantitative urine protein measurement is essential for accurate CKD diagnosis and optimal patient management, facility-specific measures, including promoting inter-departmental collaboration with nephrology departments and standardizing CKD diagnostic protocols across clinical specialties, should be considered critical strategies to facilitate early CKD detection and intervention. Although the data analyzed in this study were collected before SGLT2i were approved as therapeutic agents for CKD in Japan, these agents have since been introduced and are increasingly used in clinical practice. At later stages of CKD, the benefit of early nephrology involvement is well established for preparation of renal replacement therapy^11,12^. Therefore, the importance of early diagnosis and prompt intervention is expected to become even greater.

## Supplementary Information

Below is the link to the electronic supplementary material.

Supplementary Material 1

Supplementary Material 2

## Data Availability

The datasets generated and/or analysed during the current study are not publicly available due to JMDC Inc.‘s policy, but are available from the corresponding author on reasonable request.
