# Supplementary material for: Management status of patients with chronic kidney disease across medical specialties in Japan: a real-world data analysis
Source: Sci Rep. 2025 Dec 8;16:1536. doi: 10.1038/s41598-025-31735-2 (PMC12796421; doi:10.1038/s41598-025-31735-2)
Supplement: Supplementary file 2 — Supplementary Material 2 [file 41598_2025_31735_MOESM2_ESM.pptx]

## Slide 1
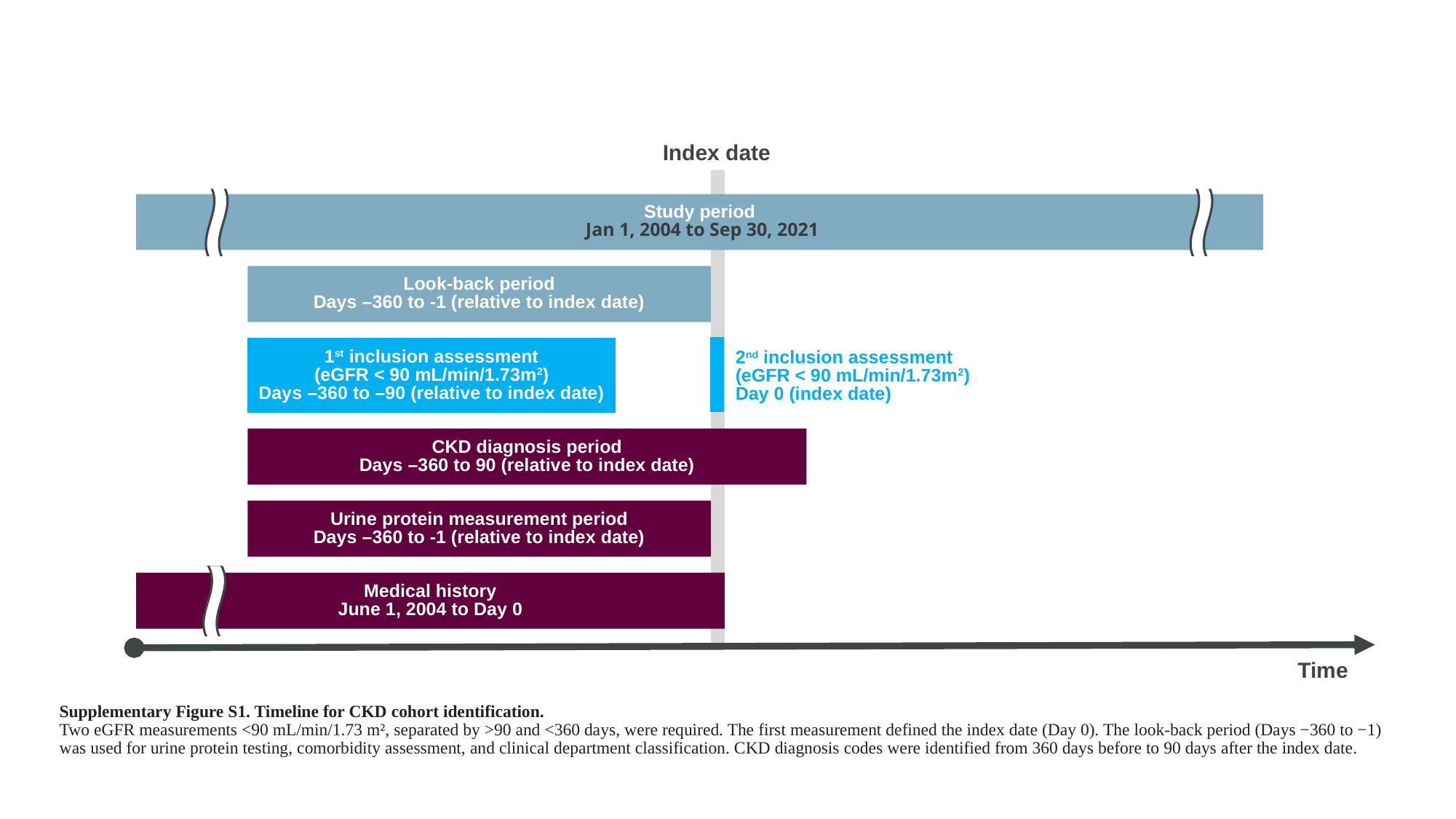

Index date
Study period
 Jan 1, 2004 to Sep 30, 2021
Look-back period
Days –360 to -1 (relative to index date)
1st inclusion assessment
(eGFR < 90 mL/min/1.73m2)
Days –360 to –90 (relative to index date)
2nd inclusion assessment
(eGFR < 90 mL/min/1.73m2)
Day 0 (index date)
CKD diagnosis period
Days –360 to 90 (relative to index date)
Urine protein measurement period
Days –360 to -1 (relative to index date)
Medical history
June 1, 2004 to Day 0
Time
Supplementary Figure S1. Timeline for CKD cohort identification.
Two eGFR measurements <90 mL/min/1.73 m², separated by >90 and <360 days, were required. The first measurement defined the index date (Day 0). The look-back period (Days −360 to −1) was used for urine protein testing, comorbidity assessment, and clinical department classification. CKD diagnosis codes were identified from 360 days before to 90 days after the index date.
